# Mixed Reality-Based Smart Occupational Therapy Personalized Protocol for Cerebellar Ataxic Patients

**DOI:** 10.3390/brainsci14101023

**Published:** 2024-10-16

**Authors:** Michela Franzò, Franco Marinozzi, Alessia Finti, Marco Lattao, Dante Trabassi, Stefano Filippo Castiglia, Mariano Serrao, Fabiano Bini

**Affiliations:** 1 Department of Medico-Surgical Sciences and Biotechnologies, Sapienza University of Rome, 00196 Rome, Italy; michela.franzo@uniroma1.it (M.F.); dante.trabassi@uniroma1.it (D.T.); stefanofilippo.castiglia@uniroma1.it (S.F.C.); mariano.serrao@uniroma1.it (M.S.); 2 Department of Mechanical and Aerospace Engineering, Sapienza University of Rome, 00185 Rome, Italy; franco.marinozzi@uniroma1.it (F.M.); alessia.finti@uniroma1.it (A.F.); lattao.1846351@studenti.uniroma1.it (M.L.); 3 Department of Brain and Behavioral Sciences, University of Pavia, 27100 Pavia, Italy

**Keywords:** ataxia, mixed reality, neurorehabilitation, occupational therapy

## Abstract

Background: Occupational therapy (OT) is an essential component of patient care, and it is especially beneficial if focused on meaningful activities. For ataxic patients, traditional procedures are currently the most efficient, although without specific guidelines and suggestions for virtual reality integration. In this context, this study proposes Hybrid Smart Rehabilitation (HSR) based on mixed reality (MR) as an aid in overcoming limitations of the traditional OT procedures. Methods: MR-HSR is designed specifically for ataxic patients and developed in Unity with the Holographic Remoting setting for run-time intervention on the scene. The subject reaches a book and grabs it with their hand inside a holographic guide with audio-visive feedback. Hand trajectories acquired from eight ataxic patients and eight healthy subjects were compared and new variables were analyzed to evaluate the performance. The Trust in Automation questionnaire was submitted to assess the opinion of the patients. Results: Patients confirmed their trust in the developer and in the improvement that this system can bring to their rehabilitation. The “total time” and “sway area” of the trajectory were statistically significant and, together with the deviation of the trajectory from the main axis of the guide, although not statistically significant, made it possible to build a classifier. Conclusions: The patient-specific MR-HSR can be considered as an integrative tool for assessing the subject’s condition by analyzing new quantitative variables which, if matched to the Scale for the Assessment and Rating of Ataxia (SARA), could be the basis of a new index to assess the progressiveness of ataxia.

## 1. Introduction

Cerebellar ataxias are categorized as rare diseases according to the European Medicine Agency (EU/3/18/2059) because they affect approximately 0.2 in 10,000 people in the European Union. This range is extremely below the ceiling for orphan designation set to 5 people in 10,000. Cerebellar ataxia is a heterogeneous and complex group of disorders characterized by motor and non-motor symptoms that are still a challenge for clinicians and researchers.

Although the level of evidence of articles on rehabilitation of patients with ataxia are not usually high, rehabilitation—including motor and respiratory physical therapy, speech therapy, and occupational therapy (OT)—is an essential component of patient care and it is considered beneficial in the long-term [1]. OT is a group of activities adapted to improve the function, capacity, and participation of the patient. According to the Occupational Therapy Europe Association, OT aims to support “persons’ engagement in occupations and activities that they want, need and choose to do in their everyday life”. No guidelines are provided specifically for the treatment of diseases like cerebellar ataxias. In fact, the guidelines in refs. [2,3] provide only advice on interventions for OT based on scientific research and literature reviews such as promoting normal posture and movement, advice on activities of daily living, and improving proximal stability and automatic equilibrium. They also suggest task-orientated and client-centered strategies combined with compensatory equipment and techniques that limit degrees of movement and dampen tremor including postural stability, splinting, and assistive technology. The shared suggestion is that OT should focus on activities that are important and meaningful to the person. This aspect is particularly important for patients with neurological diseases, where OT is used to identify the impact and performance of the patient breaking down individual tasks into their component parts [4].

Other than OT approaches, researchers are studying specific symptoms of ataxia concerning motor abilities. The goal is to identify definite gait patterns [5,6,7] or fall risk parameters [8,9]. Constant high-intensive motor training is still the main process for the management of spinocerebellar ataxia (SCA). However, some studies assess that videogames and virtual reality enhance balance [10,11], coordination [12,13,14,15], and postural control [16]. This is especially true if combined with specific motor programs aiming at preventing falls and improving mobility, resistance, posture, balance, and muscular strength [17].

Over the last decade, several examples of applications of virtual and augmented reality in rehabilitation have been proposed. Videogames developed for health purposes are named exergames. They can be based on different technologies and devices, i.e., 2D monitors, headsets, controllers, Inertial Measurement Units (IMUs), and depth cameras. Many reviews [18,19,20,21] report that new technologies are increasingly being applied in the rehabilitation field. Furthermore, exergames have been shown to lead to real improvements for patients compared to traditional therapies. In particular, the innovative aspect consists of the playful component of the new therapy which encourages patients to carry out otherwise less interesting rehabilitation activities.

One of the latest innovative technologies is mixed reality (MR), which differs from virtual reality because it does not require any fictitious environment. MR allows for the overlapping of holograms in the real surrounding world. Moreover, it differs from augmented reality since it allows the user to interact with holograms directly with their hands. MR has been used in different contexts of medicine, though the accuracy in hologram generation and hand tracking is still low [22,23]. In [21], a review of MR rehabilitation programs and systems is provided, investigating several aspects, i.e., software and hardware components, improvements in physical abilities, and characteristics influencing effectiveness. Also, ref. [21] shows that, in rehabilitation, MR rehabilitation systems can improve physical capabilities and the game factor is fundamental. However, augmented reality is more studied than MR. Moreover, rehabilitation-specific exergames are studied more frequently than videogames used for general purposes. Programs using monitors are more frequently used than head mounted display (HMD). So, specific exergames should be implemented for each category of rehabilitative protocols to provide exercises useful to treat patients. Of main importance are rehabilitative procedures for neurogenerative diseases, such as cerebellar ataxia.

In a context where cerebellar ataxia is a rare disease, traditional procedures, as OT, are currently the most efficient specific guidelines, and the most commonly suggested technology to be integrated is virtual reality, with a 3D HMD or a monitor. The research questions the authors would like to answer are as follows: What are the limitations of traditional procedures of occupational therapy? Can Hybrid Smart Rehabilitation (HSR) based on MR be a valid aid in overcoming these limitations and producing better results?

Individuals with cerebellar ataxia have impaired ability to adapt to new motor learning patterns, relative to previous experiences. Cerebellum disease can manifest in tremors, poor and inaccurate coordination, and irregular movements. These symptoms can lead to dysmetria and deficits in grasping [24]. One of the clinical tests to evaluate these symptoms is the Finger-to-Nose test [25]. This test consists of asking the patient to touch the examiner’s finger, about an arm’s length away, and then his or her own nose. Then, the patient is asked to repeat the action more quickly or the target finger could be moved to increase the difficulty of the task. Reinforcement feedback learning has been demonstrated to be an alternative motor learning strategy in cerebellar ataxia. In this approach, activities should reinforce actions leading to a successful outcome and avoid unsuccessful actions [26]. Considering these suggestions and specific needs of ataxic patients according to their Scale for the Assessment and Rating of Ataxia (SARA) evaluation, the aim of this study is to propose an HSR protocol based on MR. The protocol uses MR as an open, smart, and flexible technology. Existing virtual reality systems used currently in clinics or proposed by researchers consist of different exercises, although with a limited number of parameters to set. Therefore, the same settings are applied across all categories of patients. Instead, HSR technology can integrate in the conventional procedure features like quantitative evaluation and real-time feedback. Moreover, if properly implemented, this system is adaptable to the patient’s conditions and flexible to the patient’s needs. For example, the MR experience can be designed to be specific for ataxic patients.

In [27], a proof of concept was presented that combines the Finger-to-Nose test and MR for personalizing the neurorehabilitation of cerebellar ataxic patients. The system consists of a fanta-scientific scene where the user reaches a spaceship on a moon with their hand passing through a three-dimensional guide and grabs it. Then, the user is asked firstly to take the spaceship near his or her nose and finally to pose it on another planet. The experience was designed to be flexible and adjustable, allowing for personalized treatment based on the patient’s needs and the degree of their pathology. Moreover, audio-visive feedback was provided. In [28], the hand tracking of the MR headset was explored during the reaching actions. In this approach, the environment for the rehabilitation consisted of red targets appearing in different positions in front of the user. No scenography was shown in this exercise like the one presented in [29]. The focus of [27,28] was on creating engaging and therapeutic exercises that patients can perform, contributing to their rehabilitation process. An update is proposed in this study, aiming at creating the basis of an OT exercise that can be carried out independently by a patient. Several parameters, such as audio-visual feedback and coordinates of the holographic scene, are adjustable. This flexibility allows the exergame to be personalized to the patient’s needs in relation to the degree of the pathology. Moreover, a scenography different from [27] is proposed to match the interest of older patients that may be prefer a daily environment instead of a fanta-scientific one. More scenarios can be implemented according to the patients’ preferences to make them comfortable.

The novelty of this study lies in its focus on creating a personalized rehabilitation framework that directly addresses the limitations of traditional OT methods, which lack quantitative analysis and evaluation. Unlike studies in the literature, this research proposes MR technology to enhance the rehabilitation process, allowing for real-time adaptability and responsiveness to patient feedback. In this study, data collected by the system allowed the researchers to carry out post-processing analysis and to suggest new quantitative parameters to evaluate the deficit in coordination, perception, and grasping. Thus, a deep temporal and spatial evaluation of the reaching and grabbing experience is expected to improve the efficiency of the traditional diagnostic and rehabilitative procedures. Patients’ interaction with the new OT technology was evaluated through a questionnaire that analyzes their impressions and opinions. The Trust in Automation (TiA) questionnaire [30] was selected because it is considered one of the most complete relating to the evaluation of the user’s thoughts towards technology as it takes into account several aspects (i.e., reliability/competence, familiarity, trust, understanding, and intention of developers) [31].

## 2. Materials and Methods

### 2.1. Study Participants

A total of 8 patients (age range 32–69, mean age 53.87 ± 13.69 years) voluntarily participated in the study, giving informed consent for the study. The acquisitions were conducted in Polo Pontino (ICOT, Latina) of Sapienza University of Rome. Subjects diagnosed with primary degenerative cerebellar ataxia were included. Individuals with motor impairment due to extracerebellar symptoms (spasticity, polyneuropathy, cognitive impairment (MMSE score  >  24), oculomotor abnormalities, other concomitant neurologic or orthopedic conditions, as well as visual deficits according to the Snellen visual acuity test were excluded. A control group of healthy subjects of the same age range (mean age: 53.12 ± 17.59 years) who voluntarily participated was acquired. The healthy subjects in the control group were selected according to the following criteria: age within the range, no neuromotor pathology, not in rehabilitation, no prostheses or operations undergone in the upper limbs, and no diagnosis of arthrosis. All 16 subjects (Table 1) tried the system and MR experience for the first time. No differences in age (Mann—Whitney test: *p* = 0.793) and sex (Χ^2^ test: *p* = 1.00) were found between patients and healthy subjects. SARA scale values with the fifth and sixth SARA items are also reported because they are related to the finger chase and nose–finger test.

### 2.2. System: Hardware and Software

**Figure 1:**
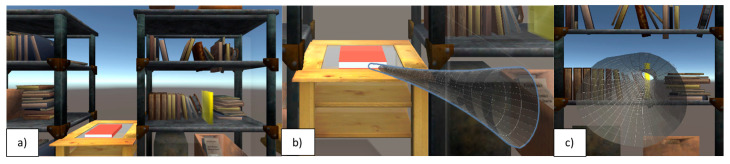
Screenshot of the Unity platform of (**a**) the scene; (**b**) the wormhole to reach the red book; (**c**) the wormhole to reach the yellow book.

The system is composed of a high-performing laptop and the MR-HMD by Microsoft (Redmond, WA, USA), HoloLens 2 (HL2). HL2, although the hand tracking accuracy can reach 2 cm [32], has been used for rehabilitative purposes in different studies [21,22]. The system proposed in this study is described in [27]. HL2 is connected by a USB to the computer, and it is managed in Holographic Remoting mode by a Unity project. The Unity project consists of a proprietary exergame developed according to the suggestions of physiotherapists and neurologists. The exergame was designed to be an exercise of occupational therapy specific for ataxic patients and patient-based. In the exergame, a scene of a holographic library is shown to the user. In front of the user and in the center of the scene, a red book is located on a table (Figure 1a). The table is by default at a 1 m distance from the subject. The Holographic Remoting mode allows the professional to modify the position of the scene according to the patient necessities (i.e., length of the arm, arm mobility). On a bookshelf, a semi-transparent yellow book is positioned. The exergame consists of two phases reproducing a pointing and reaching activity: in the first phase, the user reaches the red book on the table and grabs it (Figure 1b); in the second phase, the user brings the book on the bookshelf and poses it in correspondence with the yellow transparent book (Figure 1c). In both phases, a wormhole appears centered on the target of the reaching action: the red book in the first phase and the yellow one in the second phase. During the action of reaching the red book or placing the book on the bookshelf, the patient should insert and take their hand inside the wormhole to perform a rectilinear trajectory to the target. So, the wormhole has the role of a guide producing audio-visive feedback to the user who should adjust his/her movements. Dimensions of the wormhole can be modified by the professional in run-time, thanks to the Holographic Remoting mode, to set the difficulties of the exergame according to the troubles of the patient. The Unity app (version 2020.3.30f1) saves all the feedback generated by the interaction with a hologram and the time instants. In particular, the action of grabbing the red book is registered and the time instant is saved by the app. This action is fundamental to identify the end of the first phase of reaching and to evaluate the trajectory that the hand of the subject performed to grab the book. Considering that patients with ataxia have difficulties in perceiving distance and the volumetric position of an object, the grab activity is also challenging for them. They need to identify the correct position of the holographic book in front of them and move their hand to the volume of the book to grab it.

### 2.3. Protocol of Acquisition

**Figure 2:**
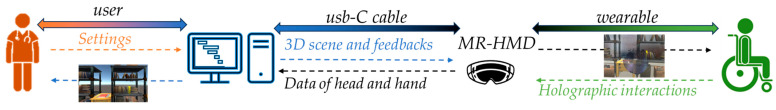
Diagram of the system workflow: from the clinician as the user, who uses the platform, sets parameters and checks the MR experience in real time, to the patient, who wears the MR-HMD and does the exercise.

Acquisitions were conducted according to the following protocol (Figure 2): (i) The laptop was positioned on a table and the HL2 was connected by a 2 m USB-c cable; (ii) the patient was seated near the table and was wearing the holographic device; (iii) the Holographic Remoting mode was activated and the exergame started; (iv) the Unity interface showed in real time the patient’s point of view, and it made it possible to intervene with the exergame for personalization (i.e., changing the distance and height of the scene, changing the size of the wormhole); (v) at the end of the exergame, the project saved data of the acquisition in several txt files. After the exergame was stopped, the questionnaire was submitted, and demographic information was collected. Each subject tried the exercise as many times as necessary (maximum 3 times) to become familiar with the instrumentation, though only the last acquisition session was considered for the analysis. For this reason, other parameters such as fatigue and the learning effect were not evaluated on this occasion. We allowed participants to perform the motor tasks with their dominant limb because we were interested in assessing the execution of an everyday-life gesture that is naturally performed through the upper limb of the dominant side.

### 2.4. Data Processing and Statistics

Data acquired by sensors integrated in the HL2 are head 3D coordinates and angles of rotations and hand 3D coordinates, angles of rotations, and velocities (30 Hz frequency). Moreover, from Unity, other data were acquired to recreate the whole experience in post-processing: wormhole 3D coordinates and dimensions, the instants of time in which exiting and entering of the hand from the wormhole occurred, red book 3D coordinates, and instants of time in which grabbing and releasing of the book were recorded.

The first phase of the exercise ended with the instance of the grab event registered by Unity, while the second phase started when the previous one ended and stopped when the collision between the red book and the yellow one was registered. The first phase of the exercise was considered for the statistical analysis. The acquired trajectories were studied on the horizontal plane (XY) in relation to the position of the red book, the position of which was recorded by Unity.

The inter-subject analysis was performed considering parameters specific for ataxic patients (jerk parameter) and new parameters based on the MR experience to evaluate different responses of the pathologic and healthy subjects. Some parameters were introduced to evaluate the volume perception of the patient, identifying the number of grab attempts and the distribution of the hand position around the book. The parameters evaluated (Table 2), other than the jerk, which is scientifically recognized for the assessment of ataxia subjects, were chosen by the authors; they are the results of experience and the need to identify valid and innovative procedures to improve the rehabilitation protocol.

The calculation of these new graphic parameters is reported in the Supplementary Materials (Figure S1).

For each parameter, a Wilcoxon Rank sum test was applied because of the not-normal distribution of the data. Principal component analysis (PCA) [34] was applied in MATLAB to the results obtained by each parameter. PCA allows one to find the combination between such parameters that explains most of the variance of the whole dataset. Then, the parameters involved in the biggest amount of explained variance are selected as the ones that contain most of the information used to classify patients versus healthy people. The pre-processing actions for PCA include the elimination of the outliers and standardization of the data [35], so the outliers were defined as points outside of the percentiles specified among 25th and 75th percentile thresholds, and the z-score method was used to standardize data. Moreover, the K-means clustering analysis was performed on data transformed in PCA space. The SVM classifier (Support Vector Machine) was trained on the original data but only including parameters with the most incidence on the two principal components that were the result of the PCA.

In conclusion, the TiA questionnaire was evaluated. Körber’s [30] questionnaire provides a total of 19 items grouped into six scales. Each scale evaluates a specific aspect of trust in automation which is consistently influenced by general propensity to trust, development experience, personality, and cultural background. For each item, an answer on a 5-point rating scale ranging between 1 (=strongly disagree) and 5 (=strongly agree) was provided. All the answers from all patients were analyzed, and the frequency of the rating point they gave for each item was evaluated.

## 3. Results

**Figure 3:**
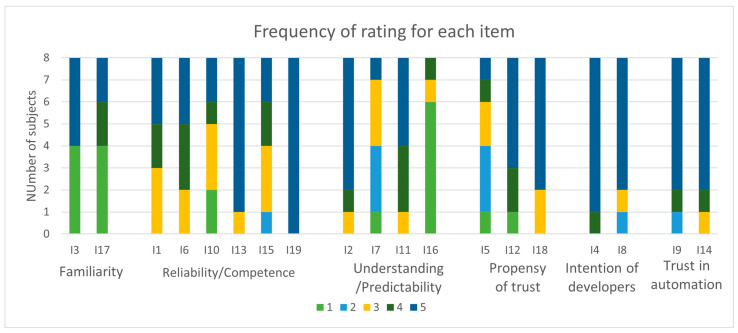
Graph summarizing the frequency of patients’ answers to each item. The items are grouped into six scales according to Körber’s instructions.

In Figure 3, the frequency of the 5-point rating scale ranging for each item is reported.

The familiarity scale shows that half of the patients were familiar with or had used similar systems. Generally, patients considered the system sufficiently reliable, and only three subjects rated the items in this category as 3 or lower, while at least two subjects rated all items 5. Among the items of the scale “understanding/predictability”, the items 7 and 16 were ranked low. These items specifically regard the predictability of the systems, so these patients had found it difficult to understand what the system would do next. Generally, patients had shown propensity and willingness to trust the automation. Moreover, the scale “intention of developers” demonstrates a trust in the developers of the system. Therefore, they recognized the usefulness of the system for their personal rehabilitation.

**Figure 4:**
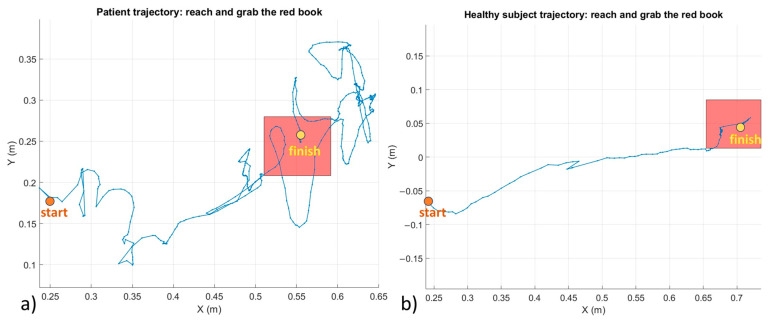
Example of hand trajectory on the horizontal plane (vision from above) during the first phase of the exercise: (**a**) patient, (**b**) healthy subject. The red square represents the book which is positioned a different distance in front of the user because of the personalization of the scenario. The blue line represents the trajectory of the hand that starts in the orange circle and stops in the green one.

In Figure 4, examples of trajectories from a patient and a healthy subject are reported. The trajectories have been truncated from the initial position and the instance of the grab event registered.

The healthy subject reaches the book through a straight curve and grabs the book by the corner or edge nearest to them. Instead, the patient draws a curve with several deviations from a straight line and their hand goes around the book sometimes before finally grabbing the book.

In Table 3, the results of the statistical test on all seven the variables studied are reported. The hypothesis was rejected only for the “sway area” and the “total time” variables with a *p*-value less than 0.05. Additional results are reported in the Supplementary Materials (Figures S2 and S3).

**Figure 5:**
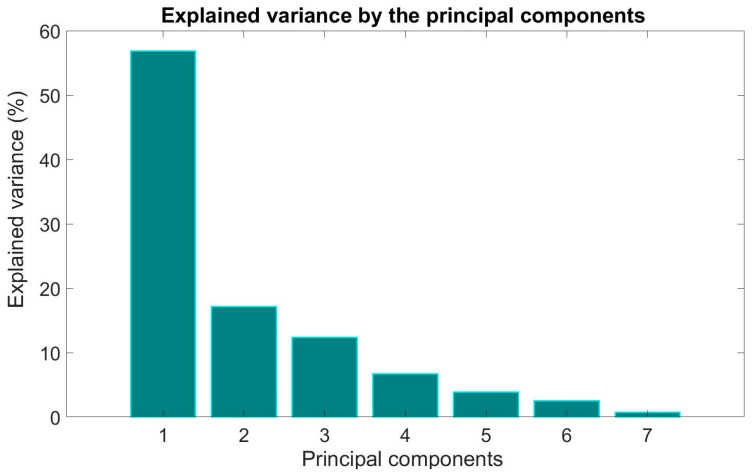
Variance explained by the principal components (PCA). PC1 accounted for 59.79% of the total variability, while PC2 explained 17.16% of the total variability.

**Figure 6:**
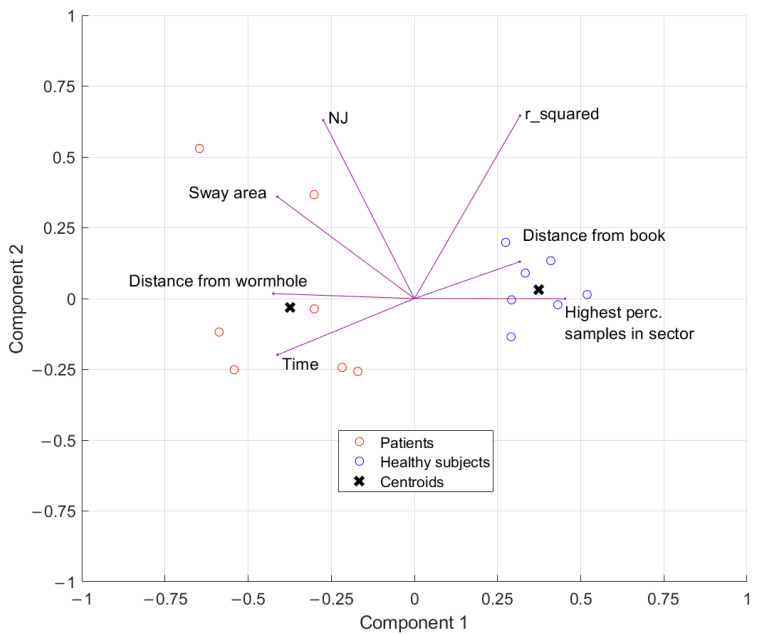
Principal component analysis (PCA) biplot depicting the relationship between the estimated variables. The contribution of traits to PC1 and PC2 is indicated by the length of the arrows. The circles are the original data points projected into the PCA space. The different colors represent the two clusters of 8 subjects each. The black crosses identify the centroid of the two clusters identified by the K-means algorithm.

In Figure 5, the number of principal components obtained by the PCA and the variance explained by them is shown. Considering that the first component of the PCA explained more than half the variance, Figure 6 presents the biplot of the two principal components resulting from the PCA and the respective clustering.

**Figure 7:**
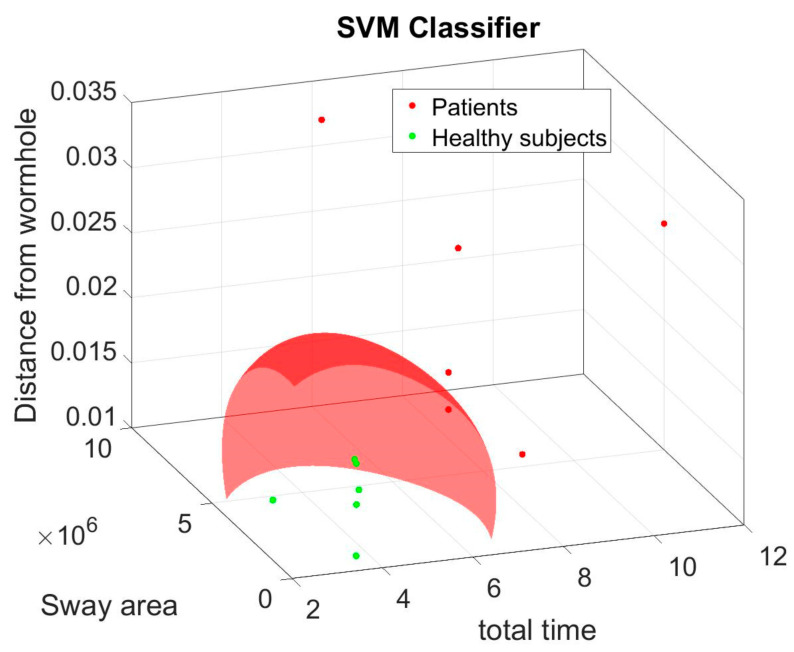
Three-dimensional graph of the SVM classification based on the three variables selected according to the interpretation of the biplot of the PCA among the variables with more incidence on the PC1.

The total number of data points, plotted in the scatter plot (Figure 6), is less than the original number of subjects. This reduction is caused by the pre-processing of the data for the PCA. In particular, it is due to the elimination of outliers. From the interpretation of the incidence of each variable on PC1 [36], “time”, “sway area”, and “mean distance of the trajectory from the wormhole axis” turned out to be the variables that produced the most of the variance in the dataset. Thus, these variables were selected as the most representative of separation between healthy subjects and patients. The normalized jerk factor (NJ) and the “R_Square” variables were not included in classifier training because they stood in the middle of the two data point clouds. Eventually, the remaining variables were not included because they stood in the healthy group data point cloud. The original data belonging only to the selected variables were used to train a classifier. The result of the SVM classification based on the three variables is reported in Figure 7, showing the separation between healthy subjects and patients. To train the classifier, the original dataset was divided into a training and a test set according to percentages of 70% and 30%. The classifier had a F1-score of 100%.

Table 4 shows, for each patient, the values of the three selected variables and the SARA scores.

## 4. Discussion

Data collected by the system enabled a comprehensive and reliable post-processing analysis of patients’ performances. The playful elements integrated into the occupational therapy context fostered greater patient collaboration and active participation.

From the TiA questionnaire, the analysis of the frequency of the 5-point rating scale ranging for each item (Figure 3) shows that patients had already known about or used similar systems; considered the system sufficiently reliable; had found difficulty in understanding what the system would do next; had generally shown propensity and willingness to trust the automation; trusted the developers of the system; and recognized its usefulness for their personal rehabilitation.

The analysis of the trajectories confirms the effects of ataxia on motor control by showing a clear difference in the trajectory made to reach the target and the following grasping phase. Obviously, the convolutions that the trajectory presents and the difficulty in grasping the hologram depend on the degree of advancement of the pathology, but could also be influenced by other comorbidities even in the case of healthy subjects, particularly of advanced age.

The statistical analysis of the single variables only identified “time” and “sway area” of the trajectory as statistically significant. This result was already expected if considering the motor problems of the pathology under investigation. The NJ and “R-square” parameters probably did not turn out to be significant because elderly people were also taken into consideration in both groups and comorbidities given by old age probably affected the results. On the other hand, “high percentage of sample in sector” would probably have been significant with an increase in the sample of subjects given the proximity to the threshold. Instead, the last two parameters, “distance from book” and “distance from wormhole”, were related to the perception of the holograms, so their statistical insignificance was probably related to the use of a new innovative technology. The larger number of adult subjects, less accustomed to similar games, could have caused the worse perception of the position of the holograms in space. Probably, more sessions of acquisition would lead to an elimination of this limitation for both groups.

Although the other variables were not found to be statistically significant, they were found to be incidental in Component 1 of the PCA. In fact, PCA and K-means clustering showed the separation between healthy subjects and patients. Since PCA produces a linear combination of the original variables, a variable that appeared at first not to be statistically significant could produce powerful information if coupled with other variables in PCA space. Moreover, the SVM classifier that was trained by including only original data of the most meaningful parameters according to PCA confirmed the separation between the two classes of patients and healthy subjects. Clearly, the future deepening of the dataset will add more value to such considerations. The high age of the sample should be taken into account. In fact, although the oldest subject (80 years) was present in the control group, the age range was more dispersed, unlike the group of patients in which age was more concentrated around the middle value. Despite this, the classifier still managed to distinguish the two groups.

In fact, although “time” and “sway area” were confirmed as the most incident variables, other variables also showed comparable incidences, meaning that a combination between them and the variables could provide even more information. These parameters can be evaluated only with this methodology and technology, and they have been demonstrated to be meaningful in describing the behavior of the subjects in terms of the perception of the 3D object and the capacity to follow a linear trajectory towards the object. Consequently, these variables can be considered as the basis of new indexes to diagnose the pathology and to evaluate its score of progress. This deduction is corroborated by observing the values summarized in Table 4. This last one shows that patients with the highest SARA 5 score equal to 3 regarding the finger chase action record either a longer time with a corresponding low “sway area” and distance from wormhole or, conversely, a low time but higher values of the other parameters. From these results, it can be deduced that subject 7 moved their hand with impulsive and fast gestures; in fact, he/she covered a greater area in a shorter total time. Meanwhile, subject 2, who was diagnosed with the highest level of the sixth item, recorded the highest values of “sway area” and distance from wormhole in a longer time. This suggests a better perception of space, but less motor control. No more in-depth statistical analysis was performed due to the small size of the analyzed sample.

The findings of this study support and build on previous research, showing that virtual and augmented reality technologies are effective in rehabilitation. For instance, IMU-based balance assessments turned out to be reliable for clinical stroke rehabilitation [10], while [18,19] assessed human pose estimation in the clinical context. Our study also confirms that this technology for hand tracking works well for different conditions. Similarly, the review in [11] showed that balance and coordination training for patients with genetic degenerative ataxia found benefits from these technologies, which aligns with our observations. Our study adds to this by showing similar benefits in another group using innovative hologram-based exercises. Eventually, in [21], it was highlighted that MR in rehabilitation programs may produce improved outcomes due to user excitement, enjoyment, and motivation. Our findings support this by demonstrating how elements like holograms can boost patient engagement and motor control. Our research adds to these findings by showing that mixed reality systems can effectively assess and enhance motor control related to ataxia, creating new opportunities for targeted rehabilitation interventions.

Certainly, the number of subjects could be considered a limitation to the consistency of the level of reliability. However, the rarity of the disease should be taken into consideration in evaluating the entirety of the sample. Notably, we purposefully excluded subjects with comorbidities that could further impair motor skills in addition to cerebellar damage, which increased sample homogeneity in this regard, but further reduced the available sample size. Furthermore, because of the limited sample, it was not possible to exclude the idea that the results may have been influenced by variability in motor disability within the study group through an analysis of disability subgroups. Indeed, promising results have already been obtained from alternative statistical analysis. In this way, more traditional statistical analysis approaches can be tested on a larger dataset, leading to strengthened clustering and classifier accuracy.

## 5. Conclusions

This study presents the possibility of using an innovative technology to propose different rehabilitative exercises with the suggestion of new quantitative parameters to evaluate the subject’s performance. The statistical analysis confirms that the total time of acquisition and the area covered by the movement of the hand are significant parameters to distinguish between ataxic patients and healthy subjects. Moreover, PCA was used to determine the most significant variables with which to evaluate the subjects’ performance, and the classifier built with these variables accurately discerned patients and healthy subjects. Accordingly, in a future implementation, these parameters could be considered for the development of an index to assign a score to the progressiveness of ataxia, matching it to the universally used SARA score. It is important to highlight that the parameters identified by the PCA are contextualized in the MR scene implemented and can be acquired only with this technology.

The methodology presented, comprehensive of the technology and MR exercises designed specifically for ataxic patients and accompanied by the statistical analysis presented, can be considered as an integrative tool for supporting the assignment of the score of diagnosis, for rehabilitation and for assessing a subject’s improvement.

## Figures and Tables

**Table 1:** Summary of the information regarding both the control group of healthy subjects and the patients.

N° Subject	Patients							Healthy Subjects	
	Sex	Age	Diagnosis	SARA tot	SARA 5	SARA 6	SARA 7	Sex	Age
1	F	57	Ataxia (NDD) *	12	2	1	2	M	53
2	M	40	FRDA **	32	3	2	2	F	61
3	M	57	Ataxia (NDD)	14	1	1	2	M	61
4	F	32	FRDA	6	1	0	1	F	63
5	M	69	SAOA ***	15	2	1	2	M	50
6	M	44	SAOA	21	2	1	2	F	28
7	F	67	SAOA	22	3	1	2	F	80
8	F	65	SAOA	14	2	1	2	M	29

* in determination phase, ** sporadic adult-onset ataxia (SAOA), *** Friedreich ataxia (FRDA).

**Table 2:** Parameters measured to evaluate the deficit in coordination, perception, and grasping in a reaching and grab experience.

Variables	Description	Outcomes Evaluated
Time	Total time of acquisition	Difficulty of activity
NJ	Normalized jerk factor, calculated as in [33]	Cinematic
Sway area	Total area occupied by the trajectory calculated building a polygon figure with the most external samples as the vertices (combination of “convhull” and “polyarea” MATLAB (version R2024a) functions)	Accuracy of movement (hand near the target)
R_square	Coefficient of determination; comparison between the trajectory of the hand and the ideal linear segment	Accuracy of movement (linear trajectory to the target)
Distance from the wormhole	Mean sample to sample distance of the trajectory from the principal axes of the wormhole	Accuracy of movement (linear trajectory to the target)
Highest percentage of samples in the polar sector	Movements around the target calculated as follows: (i) Considering the coordinates of the samples in polar coordinates with the position of the grab event as the center; (ii) Plotting a polar histogram to divide the samples in sectors of 30° each; (iii) Evaluating the sector with the highest percentage of samples.	Perception of the target position Dysmetria
Distance from the book	Distance between the following: The center of the distribution of samples placed inside the polar sector with the highest percentage of samples; The nearer point of the book that can be grabbed.	Perception of the target position Dysmetria

**Table 3:** Summary of the information regarding both the control group of healthy subjects and the patients: for each parameter, mean, standard deviation (S.D.), and *p* value are reported.

Variables	Healthy		Patients		*p* Value
	Mean	S.D.	Mean	S.D.	
Time (s)	4.66	4.51	11.05	9.71	0.0379
Normalized jerk factor (NJ) (m/s^2^)	5.17 × 10^6^	1.09 × 10^7^	1.7 × 10^7^	3.36 × 10^7^	0.4418
Sway area (cm^2^)	100	40	400	500	0.0499
R_square	0.73	0.30	0.64	0.31	0.3282
Distance from the wormhole (cm)	2.00	2.00	4.00	6.00	0.2345
Highest percentage of samples in the polar sector	78.99	19.77	56.24	25.53	0.065
Distance from the book (cm)	16.00	4.50	14.00	7.00	0.3823

**Table 4:** Time, sway area and “distance from wormhole” values compared to SARA score (final score, fifth and sixth items).

N° Subject	SARA Score		Selected Parameters		
	5	6	Time (s)	Sway Area (cm^2^)	Distance from Wormhole (cm)
1	2	1	10.57	200	1.00
2	3	2	17.90	600	4.00
3	1	1	6.02	400	3.00
4	1	0	4.43	100	1.00
5	2	1	7.73	100	1.50
6	2	1	32.27	200	1.50
7	3	1	3.41	1400	19.00
8	2	1	6.09	100	2.00

## Data Availability

The data that support the findings of this study are available on request from the corresponding author. The data are not publicly available due to privacy restrictions.

## Supplementary Materials

The following supporting information can be downloaded at https://www.mdpi.com/article/10.3390/brainsci14101023/s1, Figure S1. Graphs of the new parameters calculated for patient acquisition: (a) Distance from the wormhole principal axes; (b) sway area; (c) sector with the highest percentage of sample; (d) center of the distribution in the sector with the highest number of samples to calculate its distance from book. Figure S2. Boxplots of each variable evaluated for both groups of patients and healthy subjects. Table S1. Mean and standard deviation of the hand rotations and linear velocity for each subject. Figure S3. Boxplot of mean value of rotations and linear velocity for patients and healthy subjects.
