# Supplementary material for: Mixed Reality-Based Smart Occupational Therapy Personalized Protocol for Cerebellar Ataxic Patients
Source: Brain Sci. 2024 Oct 16;14(10):1023. doi: 10.3390/brainsci14101023 (PMC11506775; doi:10.3390/brainsci14101023)
Supplement: Supplementary file 1 [file brainsci-14-01023-s001.zip › brainsci-3237129-supplementary.pdf]

SUPPLEMENTARY MATERIALS

In this Supplementary file in-depth analysis of the method of calculating the parameters studied and the results obtained during the data analysis are reported.

The parameters are:

- **Time**: total time of the exercise, from the first instant of appearance of the hand in the field of view and the instant when the book was grasped.
- **Normalized Jerk**: calculated through the formula

$$NJ_i = \sqrt{\frac{1}{2} * \int j_i^2(t) * \left(\frac{duration^5}{lenght^5}\right) dt}$$

  where:
  - i is the coordinate (x,y or z);
  - j is the jerk calculated as $j_i = \frac{diff(acceleration_i)}{diff(tempo)}$
  - duration [s] is the total time
  - length [m] is the total space of the trajectory
- **R square**: coefficient of determination (R^2), calculated starting from a least squares approach using functions 'polyfit' and 'polyval' from Matlab to fit a polynomial of appropriate degree for each dimension. For each dimension a coefficient of determination was calculated:

$$R^2 = 1 - \frac{RSS}{TSS} \text{ ; } RSS = Residual\ Sum\ of\ Squares = \sum_{i=1}^{n} e_i^2 = \sum_{i=1}^{n} (y_i - \hat{y_i})^2 \text{ ; } TSS = Total\ Sum\ of\ Squares = \sum_{i=1}^{n} (y_i - \overline{y_i})^2$$

  where y_i is the original data, $\hat{y_i}$ is the estimated data and $\overline{y_i}$ is the mean of all original data. Finally, the mean value was calculated to obtained a single coefficient of determination.
- **Distance from wormhole** (Figure S1a): the position of the wormhole in the holographic scene is saved by the system, so the position of the principal axes of the wormhole can be represented in the same graph of the trajectory and the book. The distance of the trajectory from the axes of the wormhole was calculated as the mean of all distances of sample from the line:

$$d = \frac{|(x_2 - x_1)(y_1 - y_p) - (x_1 - x_p)(y_2 - y_1)|}{\sqrt{(x_2 - x_1)^2 + (y_2 - y_1)^2}}$$

- **Sway area** (Figure S1b): the convex polyline that contains the trajectory was determined with the function 'convhull' from Matlab, followed by the function 'polyarea' to calculate the area inside the polyline.
- **Percentage of samples in polar sectors** (Figure S1c): polar representation of the samples of the trajectory, where the centre of the graph is the geometrical centre of the book, the 0° is the horizontal axes and the radius is the distance between the sample and the centre. The polar graph was divided in 12 sectors of 30° each and the number of samples in each sector was evaluated. The polar representation was made using the functions 'polarplot' and 'polarhistogram' by Matlab.
- **Distance from the book** (Figure S1d): from the polar representation centred in the geometric centre of the book, the sector with the highest number of samples was selected and among these samples the centre of the distribution was calculated. Finally, the minimal distance between the centre of the distribution and the geometrical perimeter of the book was

calculated, to determine how much distanced was the hand of the user from the nearer point of the target.

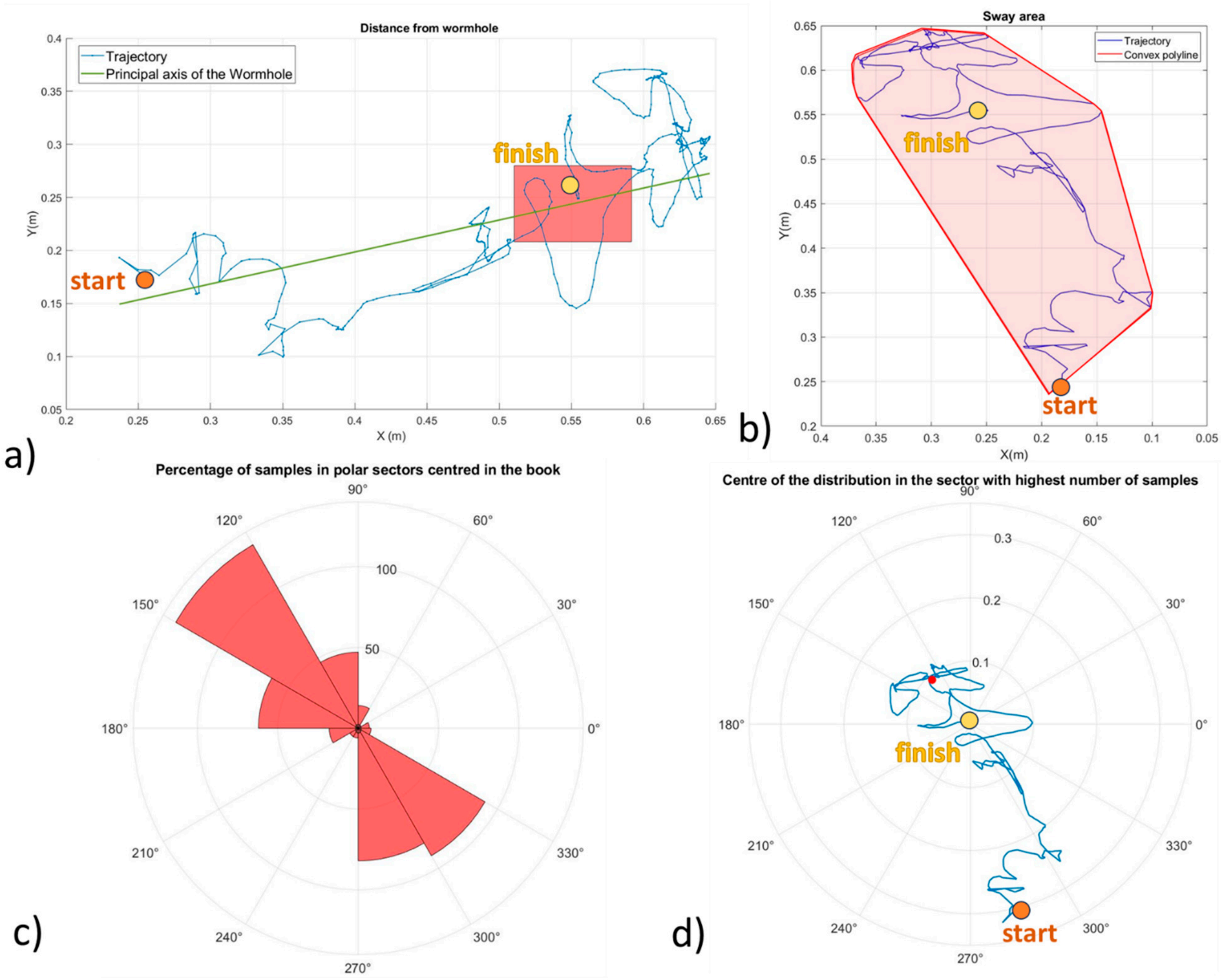

Figure S1. Graphs of the new parameters calculated for a patient acquisition: a) Distance from the wormhole principal axes; b) Sway area; c) sector with the highest percentage of sample; d) centre of the distribution in the sector with highest number of sample to calculate the its distance from book.

In figure S2 all the boxplots of each variable studied are reported to show the distribution of each samples on which the statistic test was applied to evaluate the statistical significance of the variables.

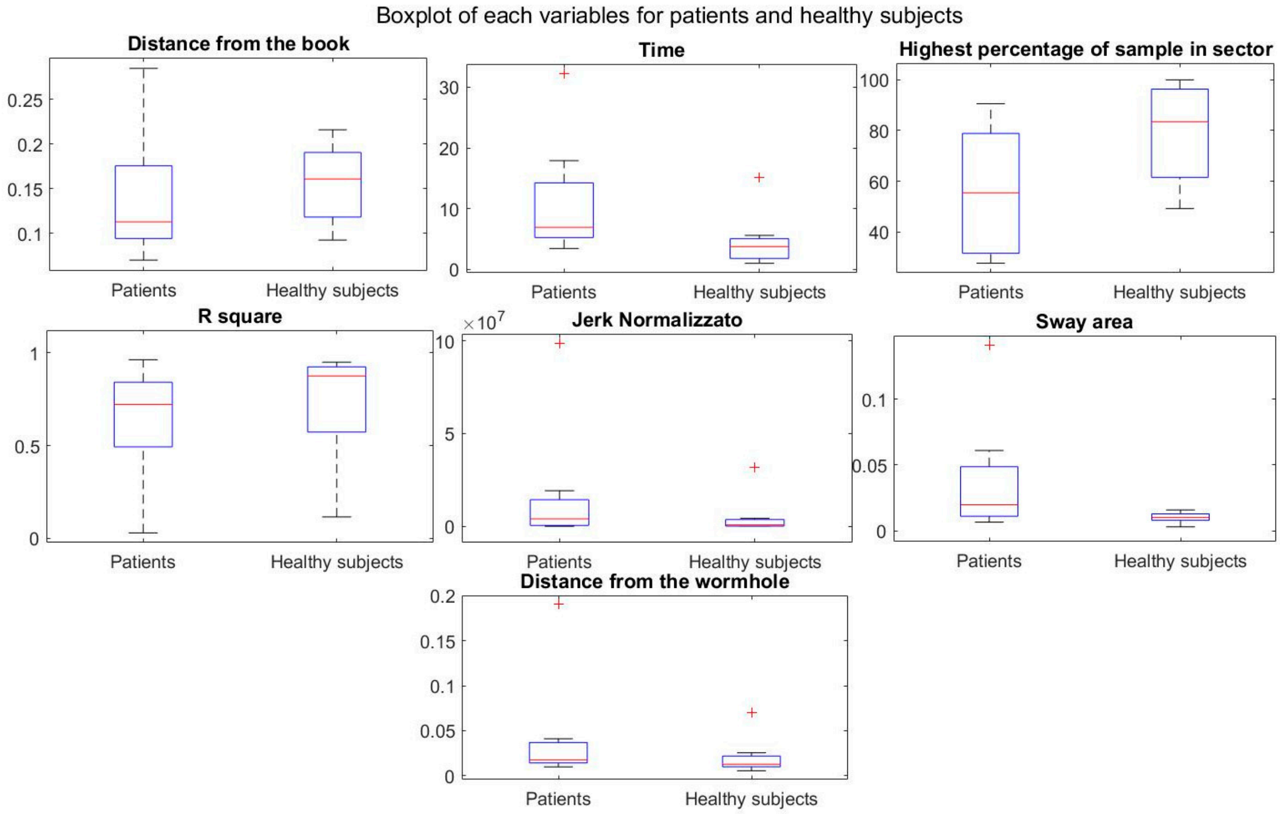

Figure S2. Boxplots of each variable evaluated for both groups of patients and healthy subjects.

HoloLens acquired, other than the coordinates of the position of the hand in time, the three axes rotations and linear velocity. These data are recorded and calculated by the proprietary algorithm integrated in the HoloLens for the hand tracking. For each subject the mean of the rotations and linear velocity values was calculated and reported in table S1, while in Figure S3 the boxplots are reported to summarise the information and comparing the two group of subjects.

Table S1. Mean and standard deviation of the hand rotations and linear velocity for each subject.

		Rot_x		Rot_y		Rot_z		vel_x		Vel_y		Vel_z	
	subjects	media	std	media	std	media	std	media	std	media	std	media	std
healthy	1	28.17	12.99	-40.82	16.93	-124.41	158.91	0.16	0.17	0.07	0.06	-0.04	0.15
	2	88.88	127.33	-56.78	16.76	-126.31	146.85	0.09	0.08	0.02	0.04	-0.04	0.12
	3	28.78	8.04	-50.03	68.09	-171.64	169.65	0.09	0.05	0.03	0.02	0.00	0.02
	4	34.08	7.28	-46.08	33.01	-31.95	57.80	0.02	0.04	0.01	0.03	0.00	0.02
	5	341.95	19.39	-28.77	19.94	-322.21	28.84	0.16	0.15	0.10	0.10	-0.19	0.29
	6	15.26	19.82	-39.04	61.54	-286.59	138.46	0.08	0.13	0.02	0.04	-0.01	0.03
	7	5.23	2.96	-22.75	10.98	-332.95	5.03	0.08	0.06	0.02	0.03	0.00	0.01
	8	195.27	175.22	-230.94	160.64	-19.21	5.42	0.11	0.24	0.00	0.02	-0.06	0.04
patients	1	325.72	69.49	-70.06	96.21	-299.07	85.12	0.03	0.03	0.00	0.04	-0.02	0.11
	2	35.77	25.11	-68.22	94.80	-63.58	103.68	0.02	0.04	0.00	0.06	0.01	0.05
	3	115.51	152.61	-37.93	17.72	-317.42	42.90	0.05	0.11	0.04	0.07	-0.06	0.18
	4	37.13	93.63	-22.72	9.66	-122.22	163.51	0.04	0.03	0.01	0.01	0.01	0.02
	5	24.16	8.09	-39.95	14.06	-160.81	171.94	0.04	0.03	0.02	0.02	0.00	0.01
	6	149.55	167.25	-43.44	12.82	-332.99	6.69	0.01	0.03	0.01	0.02	0.00	0.02
	7	255.00	110.11	-82.95	122.96	-285.85	107.95	0.04	0.08	0.09	0.18	-0.08	0.11
	8	53.62	119.74	-108.90	119.56	-323.07	69.16	0.05	0.08	0.02	0.02	0.00	0.03

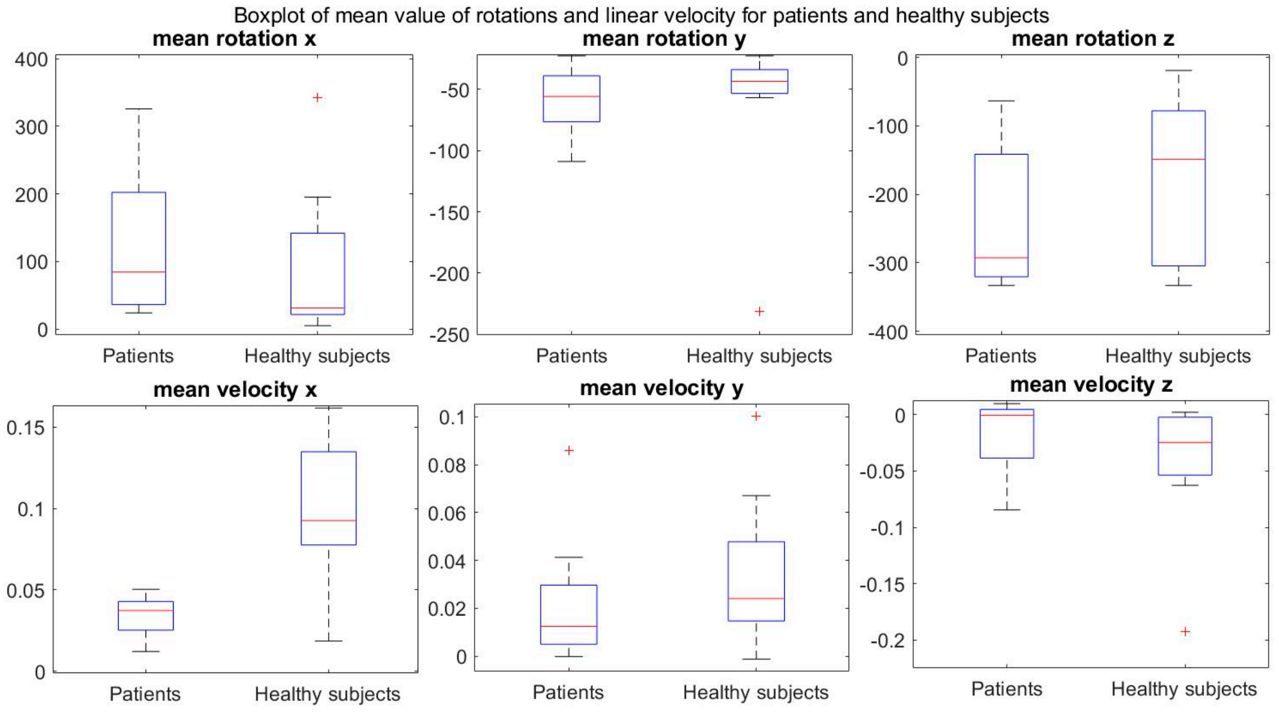

Figure S3. For each subject, the mean of the rotation and linear velocity values for each coordinates were calculated and the boxplots for both groups of patients and healthy subjects are here reported.
